# Good governance and good health: The role of societal structures in the human immunodeficiency virus pandemic

**DOI:** 10.1186/1472-698X-5-4

**Published:** 2005-04-25

**Authors:** Anatole S Menon-Johansson

**Affiliations:** 1 John Hunter Clinic, St. Stephen's Centre, Chelsea & Westminster Hospital, London, SW10 9NH, UK

## Abstract

**Background:**

Only governments sensitive to the demands of their citizens appropriately respond to needs of their nation. Based on Professor Amartya Sen's analysis of the link between famine and democracy, the following null hypothesis was tested: "Human Immunodeficiency Virus (HIV) prevalence is not associated with governance".

**Methods:**

Governance has been divided by a recent World Bank paper into six dimensions. These include Voice and Accountability, Political Stability and Absence of Violence, Government Effectiveness, Regulatory Quality, Rule of Law and the Control of Corruption. The 2002 adult HIV prevalence estimates were obtained from UNAIDS. Additional health and economic variables were collected from multiple sources to illustrate the development needs of countries.

**Results:**

The null hypothesis was rejected for each dimension of governance for all 149 countries with UNAIDS HIV prevalence estimates. When these nations were divided into three groups, the median (range) HIV prevalence estimates remained constant at 0.7% (0.05 – 33.7%) and 0.75% (0.05% – 33.4%) for the lower and middle mean governance groups respectively despite improvements in other health and economic indices. The median HIV prevalence estimates in the higher mean governance group was 0.2% (0.05 – 38.8%).

**Conclusion:**

HIV prevalence is significantly associated with poor governance. International public health programs need to address societal structures in order to create strong foundations upon which effective healthcare interventions can be implemented.

## Background

It has been argued that famine only occurs in nations that are immune to the political will of their people [1]. Political freedom in famine free countries is additionally coupled, albeit unevenly, to other freedoms such as education, health, the control of family size and the ability to seek employment.

Relatively recently, global institutions such as the World Health Organization (WHO) and the World Bank have made the link between macroeconomics and health [2,3]. Analysis of poverty around the world highlights those countries that are 'very unlikely' to meet the World Bank groups' millennium development goals (MDGs) [4]. These MDGs include the combat of HIV/AIDS, malaria and other diseases, improvement in maternal health, achievement of universal primary education, promotion of gender equality and empowerment of women, reduction in child mortality and the eradication of extreme poverty and hunger.

Some of the shared societal structures underpinning economic growth and health are the absence of violence, government effectiveness, the rule of law, lack of corruption and the ability to select a government. Even though all of these are clearly desirable the relative weight of each societal structure necessary for a strong nation state is debatable [5]. The risk of infectious disease is determined not only by pathogens and the response of the patient but also by powerful societal forces that override individual knowledge and choice [6]. Paul Farmer has coined the phrase 'structural violence' that reflects the limit of life choices, particularly of women, by racism, sexism, political violence, and grinding poverty.

The 2004 World Health Report discusses the challenges of tackling the HIV pandemic [7]. In the African continent, HIV is implicated in poor economic performance and falling gross domestic product (GDP). Within this document it describes the wide range of international support garnered to meet this challenge. However, even though the requirement of local and national government co-operation is stressed within this document, it does not elaborate on the massive heterogeneity inherent within this mandatory component.

In order to investigate the strength of the relationship between the quality of societal structures and the HIV pandemic, World Bank and UNAIDS sources were used to test the null hypothesis: "HIV prevalence is not associated with governance".

## Methods

A recent World Bank paper entitled Governance Matters III collated governance indicators for 199 countries / regions [8]. Governance in this document has been broken down into six dimensions that are defined in Table 1. Using these definitions, this research collected data for each country from 18 sources that are listed in Table 2. Governance data were then aggregated for each country and plotted along a continuum. Only the 2002 Governance data has been used in this paper. This dataset is available in a spreadsheet format from the World Bank website [9].

**Table 1:** Definitions of governance dimensions

**Voice and accountability**
"how those in authority are selected and replaced"
**Political Stability**
"perceptions of the likelihood that the government in power will be destabilized or overthrown by possibly unconstitutional and/or violent means, including domestic violence and terrorism"

**Government Effectiveness**
"we combine into a single grouping responses on the quality of public service provision, the quality of the bureaucracy, the competence of civil servants, the independence of the civil service from political pressures, and the credibility of the government's commitment to policies"

**Regulatory Quality**
"includes measures of the incidence of market-unfriendly policies such as price controls or inadequate bank supervision, as well as perceptions of the burdens imposed by excessive regulation in areas such as foreign trade and business development"

**Rule of Law**
"several indicators which measure the extent to which agents have confidence in and abide by the rules of society. These include perceptions of the incidence of crime, the effectiveness and predictability of the judiciary, and the enforceability of contracts"

**Corruption**
"measures perceptions of corruption, conventionally defined as the exercise of public power for private gain. Despite this straightforward focus, the particular aspect of corruption measured by the various sources differs somewhat, ranging from the frequency of "additional payments to get things done," to the effects of corruption on the business environment, to measuring "grand corruption" in the political arena or in the tendency of elite forms to engage in "state capture" "

**Table 2:** The sources used for governance data

Afrobarometer
Business Environment Risk Intelligence
Columbia University
The Economist Intelligence Unit
European Bank for Reconstruction and Development
Freedom House
Gallup International
Global Insight's DRI/McGraw-Hill
Heritage Foundation / Wall Street Journal
Institute for Management and Development
Latinobarometro
Political Risk Services
PriceWaterhouseCoopers
Reporters Without Borders
State Department / Amnesty international
World Bank
World Economic Forum
World Markets Research Center

The 2002 HIV prevalence estimates were obtained for each country. HIV prevalence is the percentage of adults aged between 15 and 49 years of age infected with HIV. One hundred and forty nine of the 199 countries / regions cited by the World Bank paper had published UNAIDS 2002 HIV prevalence estimates. Those countries / regions excluded from analysis due to the lack of HIV prevalence data are listed in Table 3. Those countries given a UNAIDS HIV prevalence estimate of < 0.1% were given a set value of 0.05%.

**Table 3:** Lists those countries that do not have HIV prevalence estimates for 2002

**Countries where UNAIDS commissioned a report but no HIV prevalence figure was published**	**Mean Governance Ranking position (1 – 199)**	**Countries / Regions where UNAIDS did not commission a report**	**Mean Governance Ranking position (1 – 199)**
Afganistan	4	Andorra	180
Albania	70	Antigua and Barbuda	146
Brunei	139	Bermuda	170
Comoros	52	Cape Verde	119
Djibouti	50	Cayman Islands	181
Gabon	86	East Timor	44
Guinea	29	French Guiana	151
Kuwait	128	Grenada	136
Lebanon	74	Kiribati	100
Liberia	6	Liechtenstein	183
Mauritania	103	Macao	138
Myanmar	5	Marshall Islands	110
North Korea	13	Martinique	157
Niger	60	Micronesia	91
Paraguay	27	Monaco	161
Qatar	135	Nauru	156
Saudi Arabia	105	Puerto Rico	166
Seychelles	126	Samoa	131
Syria	58	San Marino	171
Tunisia	114	Soa Tome and Principe	102
United Arab Emerites	152	Solomon Islands	43
		St. Kitt's and Nevis	127
		St. Lucia	129
		St. Vincents and the Grenadines	132
		Taiwan	160
		Tonga	66
		Tuvalu	172
		Vanuatu	87
		West Bank	26

In addition to separate analysis of each governance dimension, an average governance figure was obtained based on the assumption that each governance dimension was of equal importance. The null hypothesis was tested by measuring association between ranked governance and HIV prevalence data across the whole spectrum of countries (Kendall *tau *test, two tailed). Statistical analysis was performed using SPSS version 12.

Other health and economic data have been included in the Tables to illustrate the development needs of countries. The most recent maternal mortality data from the World Health Organization (WHO) data are available from the year 2000 [10]. The mean maternal mortality ratio (MMR) is the number of mothers who die per 100,000 live births. The number of physicians available per 100,000 inhabitants from 1990–2003 were obtained from United Nations [11]. Access to improved drinking water in 2002, expressed as a percentage, was obtained from WHO / UNICEF [12]. Life expectancy and the GDP per capita in US dollars corrected for purchaser power parity (GDP-PPP) were obtained from the Central Intelligence Agency World Factbook 2002 [13]. The GINI index data (1994–2001) from the United Nations are quoted to give an indication of the equity of income and resource distribution for each country [14]. A value of zero on the GINI index indicates fully equitable distribution of income and resources.

The relative investment by governments in health, education and the military is expressed as a ratio. This ratio is calculated from the percentage of GDP spent on health and education divided by the percentage of GDP spent on health, education and the military. Education expenditure from 2001 and government expenditure on health from 1999–2003 were obtained from the Human Development Report 2004 whilst military expenditure as a percentage of GDP for 2002 were obtained from the International Institute for Strategic Studies [15].

## Results

**Figure 1:**
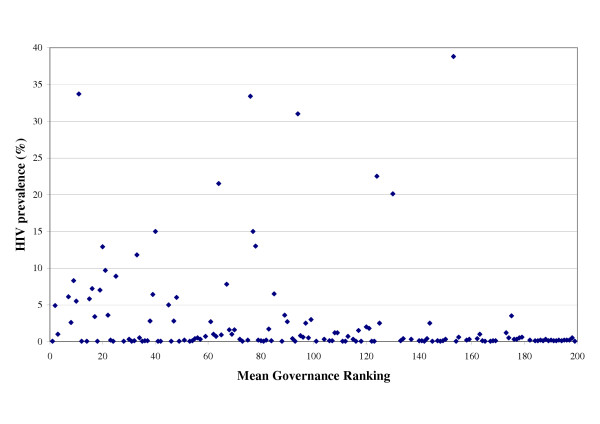
A scatter graph of Mean Governance Ranking and HIV prevalence for 149 countries.

There were fifty distinct HIV prevalence rankings from the 149 countries with UNAIDS HIV prevalence estimates in 2002. Botswana had the highest HIV prevalence estimates (38.8%) in the world that year whilst the majority of countries were placed within the lowest ranking, where HIV prevalence estimates were reported by UNAIDS to be < 0.1% (written as 0.05%). The distribution of HIV prevalence estimates by mean governance ranking is shown in Figure 1.

Non-parametric analysis of association between governance and HIV prevalence is shown in Table 4. The negative correlations indicate that HIV prevalence falls as the governance improves for each governance dimension and mean governance. The three most influential dimensions of governance were government effectiveness, the rule of law and corruption. All correlations were significant thus rejecting the null hypothesis.

**Table 4:** HIV prevalence correlations for each governance dimension and mean governance

**Governance dimension**	**Correlation coefficient (N = 149)**	**p value**
Voice & accountability	-0.123	0.032
Political Stability and Absence of Violence	-0.164	0.004
Government Effectiveness	-0.204	0.000
Regulatory Quality	-0.157	0.006
Rule of Law	-0.194	0.001
Corruption	-0.184	0.001
Mean Governance	-0.170	0.003

The dataset of 149 nations has been divided into three groups that represent the lowest (n = 50), middle (n = 50) and the highest (n = 49) governance ranking positions for each governance dimension and mean governance in Tables 5, 6 and 7 respectively. When these nations were divided into three groups, the median (range) HIV prevalence estimates remained constant at 0.7% (0.05 – 33.7%) and 0.75% (0.05% – 33.4%) for the lower and middle mean governance groups respectively despite improvements in other health and economic indices. The median HIV prevalence estimates in the higher mean governance group was 0.2% (0.05 – 38.8%).

**Table 5:** HIV, health and development data for the lowest governance ranking group.

Country Median (Range) N = 50	2002 HIV prevalence (%)	MMR (maternal deaths / 100,000 live births) in 2000	Physicians (per 100,000), 1990–2003	Improved drinking water (%) in 2002	Life Expectancy (years) in 2002	GDP-PPP ($ per capita) in 2002	GINI index 1994 – 2001	Ratio Health + Education / Health + Education + Military Spending in 2002
Voice & Accountability	1.3 (0.05 – 33.7)	525 (16 – 2000)	25 (1 – 596)	72 (22 – 100)	55.4 (37.1 – 76.4)	1621 (498 – 15650)	37.2 (26.8 – 62.9)	0.62 (0.14 – 0.91)
Political Stability	0.45 (0.05 – 33.7)	340 (7 – 2000)	27 (1 – 463)	77 (22 – 100)	63.1 (37.1 – 78.7)	2201 (498 – 18558)	37.8 (26.8 – 62.9)	0.62 (0.20 – 0.92)
Government Effectiveness	1.65 (0.05 – 33.7)	570 (7 – 2000)	23.5 (1 – 463)	71 (22 – 100)	54.5 (37.1 – 75.6)	1622 (498 – 8663)	44.1 (26.8 – 62.9)	0.69 (0.20 – 0.91)
Regulatory Quality	1.0 (0.05 – 33.7)	525 (7 – 2000)	25 (1 – 596)	73 (22 – 100)	56.6 (36.4 – 76.4)	1651 (498 – 12732)	38.2 (26.8 – 62.9)	0.62 (0.17 – 0.90)
Rule of Law	1 (0.05 – 33.7)	415 (7 – 2000)	25 (1 – 596)	74 (29 – 100)	60.4 (36.4 – 76.4)	1771 (498 – 12732)	39.6 (26.8 – 62.9)	0.68 (0.20 – 0.91)
Corruption	1.0 (0.05 – 33.7)	435 (7 – 2000)	26 (1 – 463)	75 (29 – 100)	60.8 (36.4 – 75.6)	1963 (498 – 12732)	39.3 (26.8 – 62.9)	0.70 (0.20 – 0.91)
**Mean governance**	**0.7 (0.05–33.7)**	**505 (7 – 2000)**	**25 (1 – 596)**	**73 (22 – 100)**	**59.2 (37.1 – 76.4)**	**1681 (498 – 12732)**	**37.2 (26.8 – 62.9)**	**0.64 (0.14 – 0.91)**

**Table 6:** HIV, health and development data for the middle governance ranking group.

Country Median (Range) N = 50	2002 HIV prevalence (%)	MMR (maternal deaths / 100,000 live births) in 2000	Physicians (per 100,000), 1990–2003	Improved drinking water (%) in 2002	Life Expectancy (years) in 2002	GDP-PPP ($ per capita) in 2002	GINI index 1994 – 2001	Ratio Health + Education / Health + Education + Military Spending in 2002
Voice & Accountability	0.45 (0.05 – 31)	130 (5 – 1500)	87 (2 – 463)	84 (39 – 100)	68.5 (36.4 – 79.6)	3383 (693 – 25102)	44 (28.2 – 70.7)	0.77 (0.35 – 0.94)
Political Stability	0.95 (0.05 – 33.4)	180 (10 – 1800)	68 (2 – 596)	85 (34 – 100)	65.4 (36.4 – 77.2)	3544 (675 – 35831)	44.0 (29.0 – 70.7)	0.80 (0.14 – 0.95)
Government Effectiveness	0.4 (0.05 – 33.4)	130 (2 – 1500)	90 (2 – 596)	85 (41 – 100)	69.1 (36.4 – 77.5)	3596 (675 – 12732)	40.0 (25.8 – 70.7)	0.77 (0.13 – 0.94)
Regulatory Quality	0.5 (0.05 – 33.4)	150 (5 – 1800)	85 (4 – 420)	84 (34 – 100)	66.9 (37.1 – 77.5)	3481 (770 – 10338)	44.6 (28.2 – 70.7)	0.79 (0.33 – 0.96)
Rule of Law	0.45 (0.05 – 31.0)	145 (2 – 1800)	76 (3 – 344)	84 (22 – 100)	68.0 (37.1 – 77.5)	3384 (595 – 10212)	42.2 (25.8 – 60.7)	0.77 (0.14 – 0.95)
Corruption	0.45 (0.05 – 33.4)	145 (5 – 1400)	68 (2 – 596)	83 (22 – 100)	68.5 (38.6 – 77.5)	3418 (595 – 9575)	43.0 (28.9 – 70.7)	0.77 (0.14 – 0.95)
**Mean governance**	**0.75 (0.05 – 33.4)**	**160 (5 – 1800)**	**75 (2 – 420)**	**83 (41 – 100)**	**66.9 (36.4 – 77.5)**	**3418 (693 – 9575)**	**44.6 (28.2 – 70.7)**	**0.79 (0.17 – 0.95)**

**Table 7:** HIV, health and development data for the highest governance ranking.

Country Median (Range) N = 49	2002 HIV prevalence (%)	MMR (maternal deaths / 100,000 live births) in 2000	Physicians (per 100,000), 1990–2003	Improved drinking water (%) in 2002	Life Expectancy (years) in 2002	GDP-PPP ($ per capita) in 2002	GINI index 1994 – 2001	Ratio Health + Education / Health + Education + Military Spending in 2002
Voice & Accountability	0.2 (0.05 – 38.8)	10 (0 – 730)	287 (25 – 607)	100 (24 – 100)	76.8 (37.1 – 80.8)	15961 (3085 – 35894)	34.2 (24.4 – 63)	0.86 (0.20 – 1.00)
Political Stability	0.2 (0.05 – 38.8)	10 (0 – 850)	278 (5 – 607)	100 (24 – 100)	76.0 (37.1 – 80.8)	15108 (1001 – 35894)	32.7 (24.4 – 63.0)	0.85 (0.20 – 1.00)
Government Effectiveness	0.2 (0.05 – 38.8)	10 (0 – 420)	269 (5 – 607)	100 (24 – 100)	76.9 (37.1 – 80.8)	17122 (1122 – 35894)	35.3 (24.4 – 63.0)	0.85 (0.20 – 1.00)
Regulatory Quality	0.2 (0.05 – 38.8)	10 (0 – 730)	279 (25 – 607)	100 (24 – 100)	76.9 (37.1 – 80.8)	17122 (1911 – 35894)	33.1 (24.4 – 63.0)	0.85 (0.20 – 1.00)
Rule of Law	0.2 (0.05 – 38.8)	10 (0 – 730)	269 (29 – 607)	100 (24 – 100)	76.9 (37.1 – 80.8)	17122 (1911 – 35894)	35.3 (24.4 – 70.7)	0.85 (0.20 – 1.00)
Corruption	0.2 (0.05 – 38.8)	10 (0 – 730)	269 (5 – 607)	100 (24 – 100)	76.9 (37.1 – 80.8)	17122 (1122 – 35894)	35.3 (24.4 – 63.0)	0.85 (0.20 – 1.00)
**Mean governance**	**0.2 (0.05 – 38.8)**	**10 (0 – 730)**	**279 (29 – 607)**	**100 (24 – 100)**	**76.9 (37.1 – 80.8)**	**17122 (1911 – 35894)**	**34.2 (24.4 – 63)**	**0.85 (0.20 – 1.00)**

## Discussion

It is possible to divide those nations affected by HIV / AIDS into three groups that approximate to governance ranking. The higher governance group is characterized by significant wealth and effective healthcare systems. The main challenges for these countries consists of the provision of sexual health services, health care access to marginalized groups, continuation of education and research into new and improved prevention and treatment strategies.

The HIV prevalence is generally low in higher governance group however this figure conceals differences found within specific population groups. For example in the USA, HIV prevalence amongst African American women is almost twenty three times that in whites [16]. Whilst in the UK, the prevalence of HIV amongst men who have sex with men (MSM) within London in 2001 was 100 times the national average [17]. The disparity in HIV prevalence amongst 'at risk' groups in the UK and US highlight the general difficulty of using the UNAIDS country HIV prevalence estimates. The quality of surveillance methods has been discussed and graded by UNAIDS surveillance teams, and it is clear that some HIV prevalence estimates are inaccurate [18].

Most sentinel surveillance methods use antenatal screening due to the ease of patient access and the benefits of provision of anti-retroviral treatment (ART) to prevent mother to child transmission. This surveillance strategy, though easier to implement, does not sample high risk groups such as MSM, intravenous drug users and commercial sex workers and thus underestimates the true HIV prevalence figure for the country. Within the 2002 UNAIDS HIV prevalence estimates used in this analysis there are at least four grades of surveillance. As highlighted in Table 3 some countries / regions were not included in formal UNAIDS surveillance and then there are countries where a UNAIDS report was commissioned yet no HIV prevalence estimate was provided. Reasons were not given as to why certain countries did not have a report commissioned. One fifth of the countries with UNAIDS reports quote a HIV prevalence estimate less than 0.1% and yet other health and economic indices would predict that this is an optimistic figure. Finally, there are those countries that report a HIV prevalence estimate greater than 0.1% which is complicated to varying degrees by the observer bias described above. Additional file 1 tabulates the UNAIDS HIV prevalence estimates for each of the 149 countries included in this analysis.

The governance dataset by Kaufmann *et al *in 2002 is the first global assessment of societal structures. These authors point out the variability inherent in collecting subjective material and, like UNAIDS, state the need to improve the quality of the data collected in subsequent analyses. Ideally sources would be able to freely report on each nation however, the extent of data available decreases in those nations with poorer governance. The relatively large margins of error within the governance data make direct cross-country comparisons difficult to interpret. Due to the variability of both the governance and HIV prevalence dataset the whole spectrum of data was chosen and subjected to non-parametric ranking analysis. The null hypothesis 'HIV prevalence is not associated with governance' is rejected for each dimension of governance with variations in the relative importance of different governance dimensions. Previously, Fareed Zakaria [19] has argued that democracy is less important in the development of a strong nation than the rule of law, corruption and political stability. The correlation coefficient of the voice and accountability dimension of governance with HIV prevalence was the lowest in this analysis somewhat supporting this contention.

Those countries in the lowest governance ranking group of governance are defined by poverty, ineffective health care systems, elevated HIV prevalence and significant international debt. The elevated HIV prevalence in many of these vulnerable countries was predicted more than a decade ago following the analysis of health, economic and human rights data [20]. Historically, international support has focused on short-term 'vertical' disease control strategies to tackle healthcare problems [21]. Long-term, 'horizontal' capacity building strategies are vital if HIV / AIDS is going to be effective managed in nations with limited healthcare infrastructure [22]. It has been shown in a number of resource poor settings that the provision of voluntary counselling and testing (VCT) for HIV is facilitated by the provision of free primary care services and ART [23]. The provision of effective primary care support to pregnant women is the most effective way to provide VCT services for HIV and thereby identify HIV positive mothers, prevent mother to child transmission and facilitate VCT of their partner(s). Like surveillance, this strategy though relatively effective fails to test and treat vulnerable 'high risk' groups within the population.

The poor are those most at risk of infectious disease. The role of poverty as a risk factor for disease has been clear for over 300 years [24]. Health and wealth are inextricably linked. All who become chronically ill enter a negative cycle of limited horizons. Indeed, what is true for the individual is equally true for the nation state. The effect of HIV on economic under performance and negative growth is testament to this. It is vital that essential healthcare is free, so that those that catch treatable infectious diseases are allowed to live. Encouragingly there are a few positive examples in resource poor countries, such as Uganda, Senegal and Cuba, where leadership, good communication and support of civil society have made a difference in their respective HIV epidemics. There are however many countries within this group of vulnerable nations that need the bulk of international healthcare and financial institution commitment in order to address their devastating healthcare challenges.

One example is Nigeria which is the most populous nation in Africa that has been a democracy since 1999. Despite its vast resource wealth, this country has suffered from repeated religious and ethnic conflicts that have compromised its development [25]. Only recently has the civilian government made HIV / AIDS its top priority and initiated some selective treatment programs. This change followed pressure from civil society and the military, in this latter group HIV prevalence is estimated to be 20% [26]. In contrast, South Africa is 115 mean governance ranking points higher than Nigeria, has had universal franchise for a decade and the per capita wealth is almost ten times greater than Nigeria yet the HIV prevalence is four times greater in South Africa. Some of the multiple factors that help explain the HIV prevalence in South Africa include: an earlier HIV epidemic, migration from high HIV prevalence neighbours, the violence and inequality of the Apartheid era and government inaction over the last decade. The government of South Africa has recently responded to national and international civil society pressure and has promised to provide ART free to all patients with advanced HIV disease [27].

Cuba and Haiti are islands with a similar population size and GDP-PPP per capita yet the HIV prevalence estimates are 0.05% and 6.1% respectively. HIV is thought to have entered Haiti from the USA via the sex trade in the early 1980s. The main exposure risk for Cuban nationals was from military and healthcare worker interaction with sub-Saharan Africa. Cuba was one of the first countries in the Americas to launch a nationwide HIV policy to contain transmission and care for those people living with HIV / AIDS [28]. Healthcare in Cuba is provided free to its citizens by the state and there is strong political commitment supporting health as well as national and international HIV / AIDS action. In contrast, there have been 33 coups in Haiti in the last two centuries of independence. Political instability in addition to other governance factors have been attributed to the lack of development of a responsive healthcare system [29].

As governance improves fewer women die in childbirth, more physicians exist per population, there is better access to improved water and life expectancy is longer. In addition with improvements in governance there is more GDP-PPP per capita, more equitable distribution of income and greater investment in health and education compared to the military. Interestingly, the median HIV prevalence estimates does not change between the lower and middle third groups of mean governance ranking despite a step-wise improvement in all other indices. The majority of middle ranking economies can be found within the middle governance group. Many of these countries have large populations where the HIV epidemics are set to explode. Three nations in this group who have the economic and technological power to halt their respective epidemics are Russia, China and India.

Russia has over 3 million intravenous drug users and relatively expensive ART that help to fuel the HIV epidemic [30]. The collapse of the USSR produced significant strain on the health of the people [31]. Life expectancy in Russia fell 9 years following its transition to a market economy and there has been a significant rise in 'social diseases' of Tuberculosis (TB), HIV and Hepatitis. Intravenous drug use accounts for approximately 80% of those infected with HIV however recently a new phase of the epidemic has developed that is driven by sexual transmission [32]. It is only since 2003 that there has been an increase in leadership and commitment at higher political levels to combat HIV and AIDS.

UNAIDS reported in 2002 that the number of overall infections in China increased 30% since 1998, with over 1 million people infected with HIV [33]. It is feared that China may soon experience an explosive and widespread HIV epidemic. Intravenous drug use and the sharing of contaminated needles in the south and north-west of China was one mechanism of initial transmission the other was unsafe practices among paid blood donors. Unsafe blood collections in the 1990s led to the appearance of HIV and subsequent AIDS deaths in Chinas central provinces. In response to this the Chinese authorities have recently announced that they are providing free ART in central provinces [34].

The first main focus of HIV in India was Mumbai where there is a large commercial sex work industry and the HIV prevalence reported amongst these workers is 50%. It is expected that HIV will become the largest cause of adult mortality in India in the coming decade. Despite the government making HIV its national topmost priority, any attempt to address the problem is hampered by its fractured health care infrastructure, poor literacy figures and widespread poverty [35]. At the end of 2003 the Indian government began providing free ART in eight government hospitals with the plan to expand it to a total of 25 centres [36].

The aim of this paper was to attempt to dissect out the role of governance in the HIV pandemic. It is not possible yet to determine if the relationship seen represents correlation or causation. Even though this first analysis alludes to causation, for those 149 countries with UNAIDS HIV prevalence data, the relationship will become clearer over time when it is possible to compare nations that appear similar today. Currently Brazil and India have equivalent overall governance and HIV prevalence estimates at 0.8% and 0.7% respectively. However when other health and economic indices are examined it is clear that India invest less than Brazil in health and education, has one quarter the number of physicians and double the MMR. The GDP-PPP per capita is three times greater in Brazil but it is more equitably distributed in India which is likely to contribute to equivalent life expectancy seen in both countries. India and Brazil are the main producers of generic ART. However Brazil, unlike India, has consistently provided strong political support for HIV / AIDS patients after the end of the military dictatorship in 1990. In 1996, the Brazilian government guaranteed by national law the permanent allocation of financial resources and universal access to care, including ART [37]. The current disparities between India and Brazils HIV treatment policy predicts that the Indian epidemic will progress more rapidly and is likely to impact on its development. All ten countries selected for discussion are summarised in Table 8.

**Table 8:** Ten selected countries through which the relationship of governance and HIV prevalence is discussed.

Country	Mean Governance Ranking	2002 HIV prevalence (%)	MMR (maternal deaths / 100,000 live births) in 2000	Physicians (per 100,000), 1990–2003	Improved drinking water (%) in 2002	Life Expectancy (years) in 2002	GDP-PPP ($ per capita) in 2002	GINI index 1994 – 2001	Ratio Health + Education / Health + Education + Military Spending in 2002
Haiti	7	6.1	680	25	71	49.3	1824	na	0.73
Nigeria	15	5.8	800	27	60	51.1	924	50.6	0.31^b^
Cuba	53	0.05	16	596	91	76.4	1717	na	0.79
Russia	65	0.9	45	420	96	67.3	7699	45.6	0.59
China	84	0.1	28	164	77	71.6	3535	40.3	0.33^b^
India	95	0.8	430	51	86	62.8	2136	37.8	0.65
Brazil	113	0.7	260	206	89	63.2	6477	60.7	0.77
South Africa	130	20.1	230	25	87	48.0	8466	59.3	0.85
United States	179	0.6	11	279	100	77.2	35831	40.8	0.78
United Kingdom	187	0.1	8	164	100	77.8	22801	36.0	0.82

b = no education expenditure data

na = not available

HIV / AIDS control in Russia, China and India will only be possible if they follow the example set by Brazil. International institutions need to support national civil society groups within these nations to focus the attention and resources of their respective governments for progressive healthcare changes. The global plan to stop TB outlines the possibilities and challenges that will be faced treating chronic illness, such as HIV [38]. It is pertinent that international health and financial institutions work together to influence change so that robust healthcare networks and responsive government are developed in order to apply best healthcare and economic practice.

The WHO goal of three million HIV positive persons being on ART by 2005 would be readily met if civil society in resource rich countries was able to precipitate progressive societal changes. Health is a fundamental human right, consequently each global institution, organization and citizen needs to work towards stable and progressive societal structures that can facilitate the provision of healthcare 'access for all'. The current HIV pandemic represents collective inaction and indifference towards global health. The promotion of good governance is a necessary step to enable national civil society to engineer long-term healthcare changes to deal with HIV / AIDS and future healthcare challenges.

## Conclusion

Using World Bank governance data and UNAIDS HIV prevalence estimates for 2002 this paper tests the hypothesis 'HIV prevalence is not associated with governance'. Additional health and economic indices are used to highlight the development needs for each country. The accuracy of both governance and HIV prevalence estimates are discussed and some country comparisons are made. HIV prevalence is significantly associated with poor governance. International public health programs need to address societal structures in order to create strong foundations upon which effective healthcare interventions can be implemented.

## Competing interests

The author(s) declare that they have no competing interests.

## Authors' contributions

ASM-J designed the study, performed statistical analysis and wrote the manuscript. The author read and approved the final manuscript.

## Pre-publication history

The pre-publication history for this paper can be accessed here:



## Supplementary Material

Additional File 1 Submitted Governance and Health additional file. Tabulates 149 countries by their governance ranking, HIV prevalence, health and economic data. Click here for file
