# Supplementary material for: Good governance and good health: The role of societal structures in the human immunodeficiency virus pandemic
Source: BMC Int Health Hum Rights. 2005 Apr 25;5:4. doi: 10.1186/1472-698X-5-4 (PMC1112596; doi:10.1186/1472-698X-5-4)
Supplement: Additional File 1 — Submitted Governance and Health additional file. Tabulates 149 countries by their governance ranking, HIV prevalence, health and economic data. [file 1472-698X-5-4-S1.doc]

| Country | Mean Governance Ranking 2002 | 2002 HIV prevalence (%) | MMR (maternal deaths / 100,000 live births) in 2000 | Physicians  (per 100,000),  1990-2003 | Improved drinking water in 2002  (%) | Life Expectancy in 2002  (years) | GDP-PPP $ per capita in 2002 | GINI index  1994 - 2001 | Ratio Health + Education **/** Health + Education + Military Spending in 2002 |
| --- | --- | --- | --- | --- | --- | --- | --- | --- | --- |
| IRAQ * | 1 | 0.05 | 250 | na | 81 | 66.9 | 2443 | na | a,b,c |
| Congo, Dem. Rep. (Zaire) * | 2 | 4.9 | 990 | 7 | 46 | 49.0 | 578 | na | b,c |
| SOMALIA * | 3 | 1 | 1100 |  | 29 | 46.6 | 574 | na | a,b,c |
| HAITI * | 7 | 6.1 | 680 | 25 | 71 | 49.3 | 1824 | na | 0.73^b^ |
| SUDAN * | 8 | 2.6 | 590 | 16 | 69 | 56.9 | 989 | na | 0.20^b^ |
| BURUNDI * | 9 | 8.3 | 1000 | 1 | 79 | 46.1 | 707 | 33.3 | 0.37 |
| ANGOLA * | 10 | 5.5 | 1700 | 5 | na | 38.6 | 974 | na | 0.36 |
| ZIMBABWE * | 11 | 33.7 | 1100 | 6 | 83 | 37.1 | 2481 | 56.8 | 0.74 |
| TURKMENISTAN | 12 | 0.05 | 31 | 300 | 71 | 61.0 | 4258 | 40.8 | 0.68^b^ |
| UZBEKISTAN | 14 | 0.05 | 24 | 293 | 89 | 63.8 | 2385 | 26.8 | 0.35^b^ |
| NIGERIA * | 15 | 5.8 | 800 | 27 | 60 | 51.1 | 924 | 50.6 | 0.31^b^ |
| CONGO | 16 | 7.2 | 510 | 25 | 46 | 47.6 | 1071 | na | 0.60 |
| EQUATORIAL GUINEA | 17 | 3.4 | 880 | 25 | 44 | 53.9 | 1975 | na | 0.89 |
| TAJIKISTAN | 18 | 0.05 | 100 | 212 | 58 | 64.2 | 1110 | 34.7 | 0.60 |
| SIERRA LEONE * | 19 | 7 | 2000 | 9 | 57 | 45.6 | 498 | 62.9 | 0.54^b^ |
| CENTRAL * AFRICAN REPUBLIC | 20 | 12.9 | 1100 | 4 | 75 | 43.8 | 1705 | 61.3 | 0.52^b^ |
| IVORY COAST * | 21 | 9.7 | 690 | 9 | 84 | 44.9 | 1598 | 36.7 | 0.82 |
| CHAD * | 22 | 3.6 | 1100 | 3 | 34 | 50.9 | 930 | na | 0.70 |
| LIBYA | 23 | 0.2 | 97 | 120 | 72 | 75.6 | 8663 | na | 0.61 |
| LAOS | 24 | 0.05 | 650 | 61 | 43 | 53.4 | 1597 | 37 | 0.89 |
| RWANDA * | 25 | 8.9 | 1400 | 2 | 73 | 39.0 | 875 | 28.9 | 0.61 |
| GEORGIA * | 28 | 0.05 | 32 | 463 | 76 | 64.5 | 4570 | 38.9 | 0.56 |
| BELARUS | 30 | 0.3 | 23 | 450 | 100 | 68.1 | 7613 | 30.4 | 0.71 |
| AZERBAIJAN | 31 | 0.05 | 94 | 359 | 77 | 62.9 | 3024 | 36.5 | 0.57 |
| YEMEN * | 32 | 0.1 | 570 | 22 | 69 | 60.2 | 797 | 33.4 | 0.62 |
| CAMEROON | 33 | 11.8 | 730 | 7 | 63 | 54.6 | 1645 | 47.7 | 0.84 |
| VENEZUELA | 34 | 0.5 | 64 | 200 | 83 | 73.3 | 6113 | 49.1 | 0.72^b^ |
| KYRGYZ REPUBLIC | 35 | 0.05 | 110 | 272 | 76 | 63.4 | 2651 | 29 | 0.66 |
| INDONESIA * | 36 | 0.1 | 230 | 16 | 78 | 68.2 | 2863 | 30.3 | 0.39 |
| PAKISTAN | 37 | 0.1 | 500 | 68 | 90 | 61.4 | 1950 | 33 | 0.39 |
| GUINEA-BISSAU | 38 | 2.8 | 1100 | 17 | 59 | 49.4 | 836 | 47 | 0.62 |
| ETHIOPIA * | 39 | 6.4 | 850 | 3 | 22 | 44.7 | 595 | 48.6 | 0.52 |
| KENYA | 40 | 15 | 1000 | 14 | 62 | 47.4 | 1482 | 44.5 | 0.83 |
| ALGERIA | 41 | 0.05 | 140 | 85 | na | 69.9 | 5388 | 35.3 | 0.46^b^ |
| BANGLADESH | 42 | 0.05 | 380 | 23 | 75 | 60.5 | 1546 | 31.8 | 0.75 |
| UGANDA * | 45 | 5 | 880 | 5 | 56 | 43.4 | 1092 | 37.4 | 0.69 |
| BOSNIA- * HERZEGOVINA | 46 | 0.05 | 21 | 145 | 98 | 71.7 | 1657 | na | 0.44^b^ |
| ERITREA * | 47 | 2.8 | 630 | 5 | 57 | 56.2 | 675 | na | 0.14 |
| TOGO | 48 | 6 | 570 | 6 | 51 | 54.3 | 1417 | na | 0.79 |
| IRAN * | 49 | 0.05 | 76 | 110 | 93 | 69.9 | 6245 | 43 | 0.77 |
| YUGOSLAVIA * | 51 | 0.2 | 7 | na | 93 | 73.5 | 2266 | na | a,b |
| CUBA | 53 | 0.05 | 16 | 596 | 91 | 76.4 | 1717 | na | 0.79 |
| KAZAKHSTAN | 54 | 0.1 | 210 | 345 | 86 | 63.2 | 5116 | 31.2 | 0.51^b^ |
| COLOMBIA | 55 | 0.4 | 130 | 94 | 92 | 70.5 | 6196 | 57.1 | 0.68 |
| NEPAL | 56 | 0.5 | 740 | 5 | 84 | 58.2 | 1333 | 36.7 | 0.73 |
| ECUADOR | 57 | 0.3 | 130 | 145 | 86 | 71.3 | 2822 | 43.7 | 0.62 |
| PAPUA NEW  GUINEA | 59 | 0.7 | 300 | 6 | 39 | 63.4 | 2416 | 50.9 | 0.91 |
| CAMBODIA * | 61 | 2.7 | 450 | 16 | 34 | 56.8 | 1289 | 40.4 | 0.70 |
| UKRAINE | 62 | 1 | 23 | 299 | 98 | 66.1 | 3884 | 29 | 0.75 |
| ARGENTINA | 63 | 0.7 | 54 | 304 | na | 72.2 | 12732 | na | 0.87 |
| ZAMBIA | 64 | 21.5 | 750 | 7 | 55 | 37.3 | 870 | 52.6 | 0.89 |
| RUSSIA | 65 | 0.9 | 45 | 420 | 96 | 67.3 | 7699 | 45.6 | 0.59 |
| TANZANIA | 67 | 7.8 | 1500 | 4 | 73 | 52.0 | 693 | 38.2 | 0.60^b^ |
| GAMBIA | 68 | 1.6 | 540 | 4 | 82 | 53.6 | 1063 | 47.8 | 0.88 |
| GUATEMALA * | 69 | 1 | 240 | 109 | 95 | 66.5 | 3561 | 55.8 | 0.85 |
| HONDURAS | 70 | 1.6 | 54 | 87 | 90 | 69.3 | 2654 | 59 | 0.80^b^ |
| VIETNAM | 72 | 0.3 | 130 | 54 | 73 | 69.5 | 1931 | 36.1 | 0.17^b^ |
| MACEDONIA | 73 | 0.05 | 15 | 219 | na | 74.0 | 4398 | 28.2 | 0.75 |
| MOLDOVA | 75 | 0.2 | 24 | 271 | 92 | 64.6 | 2550 | 36.2 | 0.73 |
| SWAZILAND | 76 | 33.4 | 370 | 15 | 52 | 38.6 | 3984 | 60.9 | c |
| MALAWI | 77 | 15 | 1800 | na | 67 | 37.1 | 891 | 50.3 | 0.91 |
| MOZAMBIQUE * | 78 | 13 | 1000 | 2 | 42 | 36.4 | 986 | 39.6 | 0.72 |
| ARMENIA | 79 | 0.2 | 55 | 287 | na | 66.4 | 2998 | 37.9 | 0.45 |
| BOLIVIA | 80 | 0.1 | 420 | 76 | 85 | 64.0 | 2518 | 44.7 | 0.85 |
| EGYPT | 81 | 0.05 | 42 | 218 | 98 | 63.6 | 3552 | 34.4 | 0.33^b^ |
| NICARAGUA * | 82 | 0.2 | 230 | 62 | 81 | 69.0 | 2663 | 60.3 | 0.75^b^ |
| MALI | 83 | 1.7 | 1200 | 4 | 48 | 47.0 | 827 | 50.5 | 0.70 |
| CHINA * | 84 | 0.1 | 28 | 164 | 77 | 71.6 | 3535 | 40.3 | 0.33^b^ |
| BURKINA FASO | 85 | 6.5 | 1000 | 4 | 51 | 46.4 | 978 | 48.2 | 0.55^b^ |
| TURKEY * | 88 | 0.05 | 70 | 123 | 93 | 71.2 | 6677 | 40 | 0.59 |
| BENIN | 89 | 3.6 | 850 | 10 | 68 | 49.9 | 1001 | na | 0.76 |
| GUYANA | 90 | 2.7 | 170 | 26 | 83 | 63.3 | 4877 | 44.6 | 0.92 |
| PERU * | 92 | 0.4 | 410 | 103 | 81 | 70.3 | 4475 | 46.2 | 0.79 |
| PHILIPPINES | 93 | 0.05 | 200 | 115 | 85 | 67.8 | 3742 | 46.1 | 0.77 |
| LESOTHO | 94 | 31 | 550 | 7 | 76 | 48.8 | 2343 | 56 | 0.86 |
| INDIA * | 95 | 0.8 | 430 | 51 | 86 | 62.8 | 2136 | 37.8 | 0.65 |
| EL SALVADOR * | 96 | 0.6 | 150 | 126 | 41 | 70.0 | 3848 | 50.8 | 0.89 |
| DOMINICAN REPUBLIC | 97 | 2.5 | 150 | 190 | 93 | 73.4 | 5628 | 47.4 | 0.81 |
| SENEGAL | 98 | 0.5 | 690 | 10 | 72 | 62.5 | 1556 | 41.3 | 0.81 |
| GHANA | 99 | 3 | 540 | 9 | 79 | 57.2 | 1880 | 39.6 | 0.93 |
| SRI LANKA | 101 | 0.05 | 46 | 43 | 78 | 72.1 | 3231 | 34.4 | 0.50 |
| MADAGASCAR | 104 | 0.3 | 550 | 9 | na | 55.4 | 770 | 46 | 0.72 |
| MOROCCO | 106 | 0.1 | 220 | 49 | 80 | 69.4 | 3426 | 39.5 | 0.62 |
| FIJI | 107 | 0.1 | 75 | 34 | 98 | 68.2 | 6988 | na | 0.84 |
| SURINAME | 108 | 1.2 | 56 | 50 | 92 | 71.6 | 3410 | na | 0.86^b^ |
| JAMAICA | 109 | 1.2 | 44 | 85 | 93 | 75.4 | 3639 | 37.9 | 0.93 |
| JORDAN | 111 | 0.05 | 21 | 205 | 91 | 77.5 | 3357 | 36.4 | 0.53 |
| ROMANIA | 112 | 0.05 | 33 | 189 | 57 | 70.1 | 5925 | 30.3 | 0.79 |
| BRAZIL | 113 | 0.7 | 260 | 206 | 89 | 63.2 | 6477 | 60.7 | 0.77 |
| MEXICO | 115 | 0.3 | 56 | 156 | 91 | 71.7 | 8981 | 51.9 | 0.94 |
| BHUTAN | 116 | 0.05 | 420 | 5 | 62 | 52.8 | 1122 | na | 0.73 |
| PANAMA | 117 | 1.5 | 110 | 121 | 91 | 75.6 | 5833 | 48.5 | 0.92 |
| MONGOLIA | 118 | 0.05 | 75 | 278 | 62 | 64.2 | 1770 | 44 | 0.83 |
| BELIZE | 120 | 2 | 70 | 102 | 91 | 71.1 | 3085 | na | 0.79 |
| THAILAND | 121 | 1.8 | 22 | 30 | 85 | 68.8 | 6683 | 43.2 | 0.84 |
| BULGARIA | 122 | 0.05 | 21 | 344 | 100 | 71.2 | 6228 | 31.9 | 0.61^b^ |
| CROATIA * | 123 | 0.05 | 5 | 238 | na | 73.9 | 5745 | 29 | 0.82 |
| NAMIBIA * | 124 | 22.5 | 300 | 29 | 80 | 40.6 | 4228 | 70.7 | 0.82 |
| TRINIDAD AND TOBAGO | 125 | 2.5 | 100 | 75 | 91 | 68.2 | 9575 | 40.3 | 0.95 |
| SOUTH AFRICA* | 130 | 20.1 | 230 | 25 | 87 | 48.0 | 8466 | 59.3 | 0.85 |
| MALDIVES | 133 | 0.1 | 110 | 78 | 84 | 62.5 | 1911 | na | 0.49^b^ |
| MALAYSIA | 134 | 0.4 | 20 | 68 | 95 | 71.1 | 10063 | 49.2 | 0.81 |
| BAHRAIN | 137 | 0.3 | 19 | 169 | 100 | 73.2 | 15650 | na | 0.40^b^ |
| ISRAEL | 140 | 0.1 | 11 | 375 | 100 | 78.7 | 18558 | 35.5 | 0.59 |
| OMAN | 141 | 0.1 | 87 | 137 | 79 | 72.0 | 7475 | na | 0.35 |
| SLOVAK REPUBLIC | 142 | 0.05 | 2 | 326 | 100 | 73.9 | 10212 | 25.8 | 0.83 |
| LATVIA | 143 | 0.4 | 28 | 291 | 93 | 68.7 | 7253 | 32.4 | 0.88 |
| DOMINICA | 144 | 2.5 | 730 | 49 | 97 | 73.6 | 4097 | na | c |
| KOREA, SOUTH | 145 | 0.05 | 10 | 180 | 92 | 74.6 | 15961 | 31.6 | a,b |
| LITHUANIA | 147 | 0.1 | 9 | 403 | na | 69.2 | 7312 | 36.3 | 0.70^b^ |
| POLAND | 148 | 0.05 | 9 | 220 | 95 | 73.4 | 8477 | 31.6 | 0.84 |
| MAURITIUS | 149 | 0.1 | 16 | 85 | 100 | 71.2 | 10338 | na | 0.96 |
| URUGUAY | 150 | 0.3 | 18 | 387 | 98 | 75.4 | 9226 | 44.8 | 0.86 |
| BOTSWANA | 153 | 38.8 | 100 | 29 | 95 | 37.1 | 6557 | 63 | 0.59 |
| CZECH REPUBLIC | 154 | 0.05 | 6 | 342 | na | 74.7 | 12899 | 25.4 | 0.85 |
| COSTA RICA | 155 | 0.6 | 28 | 160 | 97 | 76.0 | 6626 | 45.9 | 0.94 |
| GREECE | 158 | 0.2 | 6 | 438 | na | 78.5 | 17122 | 35.4 | 0.68 |
| CYPRUS | 159 | 0.3 | 31 | 269 | 100 | 76.8 | 13803 | na | 0.80 |
| ITALY | 162 | 0.4 | 4 | 607 | 99 | 79.1 | 22070 | 36 | 0.86 |
| ESTONIA | 163 | 1 | 42 | 313 | 87 | 69.7 | 10328 | 37.6 | 0.89 |
| HUNGARY | 164 | 0.1 | 11 | 355 | 99 | 71.6 | 11271 | 24.4 | 0.85 |
| SLOVENIA | 165 | 0.05 | 12 | 219 | na | 75.0 | 11864 | 28.4 | 0.84^b^ |
| JAPAN | 167 | 0.05 | 7 | 202 | 100 | 80.8 | 24848 | 24.9 | 0.91 |
| MALTA | 168 | 0.1 | 10 | 291 | 100 | 78.1 | 14192 | na | 0.85 |
| HONG KONG | 169 | 0.1 | na | 160 | 100 | 79.6 | 25102 | 43.4 | a,c |
| BARBADOS | 173 | 1.2 | 64 | 137 | 100 | 73.2 | 14528 | na | 0.96 |
| SPAIN | 174 | 0.5 | 3 | 329 | 24 | 78.9 | 18003 | 32.5 | 0.89 |
| BAHAMAS | 175 | 3.5 | 60 | 163 | 97 | 70.4 | 15108 | na | 0.84^b^ |
| CHILE | 176 | 0.3 | 21 | 115 | 95 | 75.9 | 9988 | 57.5 | 0.63 |
| FRANCE | 177 | 0.3 | 11 | 330 | 99 | 78.9 | 24315 | 32.7 | 0.84 |
| PORTUGAL | 178 | 0.5 | 4 | 318 | 97 | 75.9 | 15795 | 38.5 | 0.84 |
| UNITED STATES | 179 | 0.6 | 11 | 279 | 100 | 77.2 | 35831 | 40.8 | 0.78 |
| BELGIUM | 182 | 0.2 | 7 | 419 | 100 | 77.9 | 25266 | 25 | 0.90 |
| IRELAND | 184 | 0.1 | 4 | 239 | 100 | 76.9 | 21323 | 35.9 | 0.94 |
| GERMANY | 185 | 0.1 | 5 | 363 | 100 | 77.6 | 23317 | 38.2 | 0.89 |
| AUSTRIA | 186 | 0.2 | 3 | 323 | 100 | 77.8 | 24905 | na | 0.93 |
| UNITED KINGDOM | 187 | 0.1 | 8 | 164 | 100 | 77.8 | 22801 | 36 | 0.82 |
| CANADA | 188 | 0.3 | 4 | 187 | 100 | 79.5 | 24521 | 31.5 | 0.91 |
| AUSTRALIA | 189 | 0.1 | 5 | 247 | 100 | 79.8 | 23030 | 35.2 | 0.82 |
| SINGAPORE | 190 | 0.2 | 20 | 140 | 100 | 80.1 | 25532 | 42.5 | 0.20^b^ |
| NORWAY | 191 | 0.1 | 11 | 367 | 100 | 78.7 | 27557 | 25.8 | 0.88 |
| SWEDEN | 192 | 0.1 | 1 | 287 | 100 | 79.7 | 22197 | 25 | 0.89 |
| ICELAND | 193 | 0.2 | 0 | 352 | 100 | 79.5 | 24649 | na | 1.0 |
| NEW ZEALAND | 194 | 0.1 | 5 | 219 | 100 | 77.9 | 17494 | 36.2 | 0.91 |
| LUXEMBOURG | 195 | 0.2 | 18 | 254 | 100 | 77.3 | 35894 | 30.8 | 0.91 |
| DENMARK | 196 | 0.2 | 3 | 366 | 100 | 76.7 | 25445 | 24.7 | 0.91 |
| NETHERLANDS | 197 | 0.2 | 10 | 328 | 100 | 78.4 | 24303 | 32.6 | 0.87 |
| SWITZERLAND | 198 | 0.5 | 4 | 350 | 100 | 79.7 | 28421 | 33.1 | 0.92 |
| FINLAND | 199 | 0.05 | 4 | 311 | 100 | 77.5 | 22856 | 25.6 | 0.89 |
| * = war experienced since 1980 [15]  na = not available  a = no health expenditure data  b = no education expenditure data  c = no military expenditure data | | | | | | | | | |
